# High expression of EphA3 (erythropoietin-producing hepatocellular A3) in gastric cancer is associated with metastasis and poor survival

**DOI:** 10.1186/s12907-017-0047-y

**Published:** 2017-04-27

**Authors:** Baongoc Nasri, Mikito Inokuchi, Toshiaki Ishikawa, Hiroyuki Uetake, Yoko Takagi, Sho Otsuki, Kazuyuki Kojima, Tatsuyuki Kawano

**Affiliations:** 1 Matsuzawa Hospital, Setagaya-ku, Tokyo, Japan; 2 Department of Surgical Oncology, Graduate School, Tokyo Medical and Dental University, Bunkyo-ku, Tokyo, Japan; 3 Department of Translational Oncology, Graduate School, Tokyo Medical and Dental University, Bunkyo-ku, Tokyo, Japan

**Keywords:** Gastric cancer, Metastasis, Oncogenes

## Abstract

**Background:**

As the major subfamily of receptor tyrosine, erythropoietin-producing hepatocellular (Eph) receptor has been related to progression and prognosis in different types of tumors. However, the role and mechanism of EPHA3 in gastric cancer is still not well understood.

**Methods:**

Specimen were collected from 202 patients who underwent gastric resection for gastric adenocarcinoma. The expression of EphA3 was studied using immunohistochemistry. We analyzed the clinicopathological factors and prognostic relevance of EphA3 expression in gastric cancer.

**Results:**

High expression of EphA3 was associated with male predominance (*p* = 0.031), differentiated histology (*p* < 0.001), depth of tumor (*p* = 0.002), lymph node metastasis (*p* = 0.001), distant metastasis (*p* = 0.021), liver metastasis (*p* = 0.024), advanced stage (*p* < 0.001), and high HER2 expression (*p* = 0.017). Relapse-free survival (RFS) was significantly worse in patients with high expression of EphA3 than in those with low expression of EphA3 (*p* = 0.014). Multivariate analysis for RFS showed that depth of tumor [hazard ratio (HR) 9.333, 95% confidence interval (CI) 2.183–39.911, *p* = 0.003] and lymph node metastasis [hazard ratio (HR) 5.734, 95% confidence interval (CI) 2.349–13.997, *p* < 0.001] were independent prognostic factors.

**Conclusions:**

These findings suggest that high expression EphA3 may participate in metastasis and worse survival.

## Background

Although a constant decrease in gastric cancer incidence and mortality rates has been reported, stomach cancer ranks as the fifth most common malignancy and the third leading cause of death worldwide [1]. Despite current advanced therapeutic options including surgical resection, chemotherapy, hormonal therapy, radiotherapy, the estimated 5-year survival rate is still poor, varying from 64% for early stage to 4% for advanced distant metastatic stage [2]. Although many receptor tyrosine kinases (RTKs) are related to invasion and metastasis of gastric cancer, only a human epidermal growth factor receptor (HER-2) blocker has been accepted as molecular targeted therapy. Unfortunately, merely 10–20% of all patients with stomach cancer are HER-2 positive and the median survival time was only 16 months in HER-2 positive patients who underwent chemotherapy with trastuzumab [3, 4]. Hence, new diagnostic tools, novel therapeutic methods and new prognostic molecular markers for gastric cancer are urgently demanded.

Erythropoietin-producing hepatocellular carcinoma receptor (Eph) and their cell-associated ephrin ligands are associated with neoangiogenesis and invasive tumor progression, and are progressively being focused as new therapeutic targets in clinical trials [5]. Ephs and ephrins are abundantly found in multiple types of tumors, where their oncogenic roles often reflect their dichotomous developmental activities. Therefore, depending on tumor types and disease stages, high expression Ephs can stimulate or suppress tumor progression [6–8]. Recent papers have proven the prospective target therapy of EphA1 and EphA4 for gastric cancer. EphA3, a subclass of Ephs, is reported to relate to certain types of solid cancers [9, 10]. However the role and mechanism of EphA3 in gastric cancer is not well understood. We aim to elucidate the clinicopathological factors and prognostic importance of EphA3 role in gastric cancer.

## Methods

### Patients

Between January 2003 and March 2007, after excluding total 16 patients with distant metastasis at time of surgery or positive peritoneal lavage cytology for which were regarded as stage IV, there were total 202 patients undergoing gastrectomy for primary gastric tumor in the Department of Gastric Surgery of Tokyo Medical and Dental University. All participants received detailed explanation of the research, and well written informed consent was obtained. This study was designed in accordance with the Declaration of Helsinki and was authorized by the Institutional Review Board of Tokyo Medical and Dental University. All tumors were classified according to the 7^th^ edition of tumor node metastasis classification. HER2 expression was also investigated. All patients were followed up every 3–6 months with multimodalities including computed tomography, abdominal ultrasonography, endoscopy, and tumor marker analysis. Positron emission tomography and bone scintigraphy, magnetic resonance imagings were considered as needed. Patients with recurrent disease received chemotherapy with TS-1 (Titanium silicate) as single regimen or combined chemotherapy. All patients were followed up for 5 years until July 2011. The mean follow up period was 61.4 ± 25.7 months. There were 60 deaths reported with 50 (83.3%) deaths from recurrence and 10 (16.7%) deaths due to other causes.

### Immunohistocheminal Analysis of EphA3

All of the hematoxylin and eosin–stained samples were reviewed. Immunohistochemical staining was performed on 3- to 4-μM sections from formalin-fixed, paraffin-embedded tissue. After deparaffinization in xylene, the slides were rehydrated and treated with double-distilled water (DDW). Antigen retrieval by microwave pre-treatment was performed for 15 min in 6 mmol/L sodium citrate buffer (pH 6.0) (Mitsubishi Chemical Medience Corporation, Tokyo, Japan) at 98 °C. Endogenous peroxidase was quenched by 15 minutes incubation in a mixture of 3% hydrogen peroxidase solution in 100% methanol. After treating with DDW and phosphate buffered saline (PBS), specimen was incubated with the primary antibody to EphA3 (dilution 1:500) (EphA3 (L-18): sc-920 SANTA CRUZ Biotechnology, USA) for 15 min at room temperature and then 16 h at 4 °C. Specimen was treated three times with 0.1% Tween 20/PBS and then was incubated with peroxidase-labelled anti-rabbit or anti-mouse antibodies (Histofine Simplestain Max PO; Nichirei) for 30 min at room temperature. Peroxidase activity was detected with diaminobenzidine (Nichirei). Sections were then counterstained with hematoxylin.

### Interpretation of the immunostaining results

Staining intensity was classified into three grades: 0 (none), 1 (weakly positive), 2 (moderately or strongly positive). Staining extensity (positive frequency) was also classified into three grades according to the percentage of stained tumor cells: 0 (0–9%), 1 (10–49%), and 2 (50–100%). Samples with moderate positivity but staining extensity was less than 10% were graded as staining intensity of 1 (weakly positive). Composites score was the sum of the strongest intensity score and the total extensity score. For statistical analysis, composite scores ≥ 3 were classified as high expression and scores < 3 were classified as low expression. Two investigators (M.K and T.Y), who were blinded to patients’ outcomes independently evaluated stained tumor cells in at least three field per section, including the deepest site invaded by tumor cells, the most superficial site of the lesion and the intermediate zone. Any differences between the two investigators were resolved by reassessment and consensus.

### Statistical analysis

Chi-square test was utilized to analyze the hypothetical association between the expression of EphA3 and patient clinicopathological factors. Overall survival (OS), and relapse-free survival (RFS) were used to evaluate the prognosis. Kaplan-Meier curves were plotted to assess the effect of EphA3 expression on overall survival (OS) and relapse-free survival (RFS). Differences between the curves were analyzed by the log-rank test. Multivariate Cox proportional-harzards regression models were utilized to analyze the prognostic significance of expression of EphA3 and other clinicopathological factors. Statistical analysis was performed using IBM SPSS Statistics 23 software (IBM, Inc., Armonk, NY, and U.S.A). A *p* value <0.05 was considered statistically significant.

## Results

### EPHA3 immunohistochemistry

**Fig. 1:**
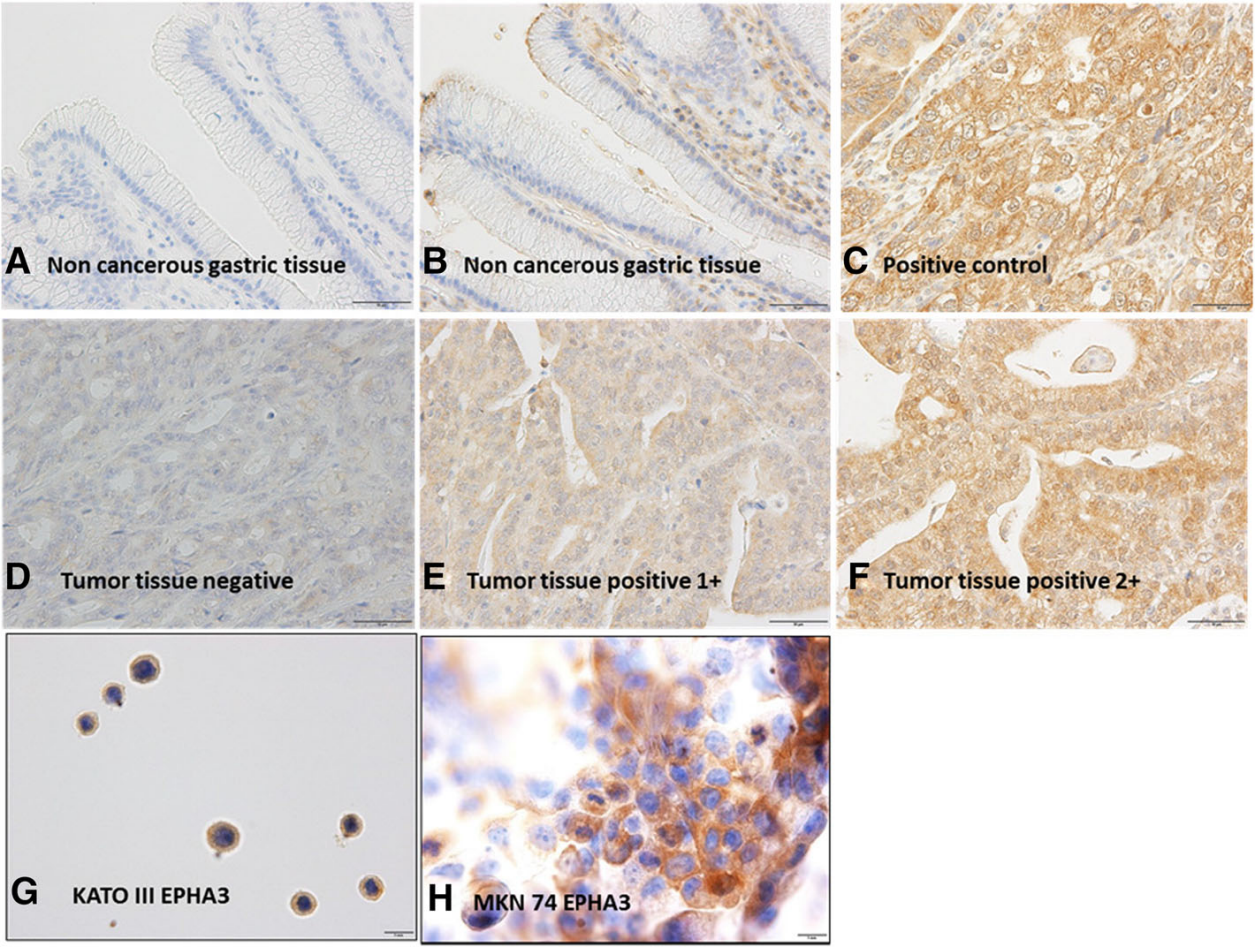
Expression of EphA3 protein in gastric cancer. **a** Non-cancerous gastric tissue which was stained without 1^st^ antibody, did not show immunostaining for EphA3. **b** Non-cancerous gastric tissue showed staining in the mesenchyme not in the mucosal layer. **c** Normal positive control showed strong immunostaining. Representative primary gastric carcinomas with intensity score of 0 (**d**), 1 (**e**), 2 (**f**). The images were captured under magnification 400x. Scale bar in the left lower corner is 50ͧͧͧͧμm. **g** KATO III EphA3 (undifferentiated type) showed weak staining. **h** MKN 74 EphA3 (differentiated type) showed strong staining

Representative cases of each staining intensity are shown in Fig. 1. EphA3 expression was mainly located in the cytoplasm and at the cell membrane. High expression (composite score ≥ 3) of EphA3 was found in 91 sample (45%) and low expression (composite score < 3) of EphA3 was found in 111 samples (55%). Non-cancerous gastric tissue which was stained without 1^st^ antibody did not show immunostaining for EphA3. Some non-cancerous gastric tissue showed staining in the mesenchyme not in the mucosal layer. We have two cell lines KATO III (undifferentiated type) which showed weak EphA3 staining and MKN 74 (differentiated type) which showed strong EphA3 staining. However staining in these cell lines is not known to express EphA3.

### Relationship between EPHA3 expression and clinicopathological factors

The association between EphA3 expression and clinicopathological factors is summarized in Table 1. High expression of EphA3 was associated with differentiated histology (*p* < 0.001), depth of tumor (*p* = 0.002), lymph node metastasis (*p* = 0.001), stage (*p* < 0.001), recurrence (*p* = 0.024), especially liver recurrence (*p* = 0.024) and HER2 expression (*p* = 0.017).

**Table 1:** Correlation between EphA3 expression and clinicopathological features in gastric carcinoma

Variables	EphA3			
	n *n* = 202	Low *n* = 111 (n, %)	High *n* = 91 (n, %)	*p* value
Age				
< 65	97	60 (61.9)	37 (38.1)	0.067
≥ 65	105	51 (48.6)	54 (51.4)	
Gender				
Female	48	33 (68.8)	15 (31.3)	0.031
Male	154	78 (50.6)	76 (49.4)	
Main location				
Middle or Lower	160	93 (58.1)	67 (41.9)	0.084
Upper	42	18 (42.9)	24 (57.1)	
WHO pathological type				
Differentiated	99	39 (39.4)	60 (60.6)	<0.001
Undifferentiated	103	72 (69.9)	31 (30.1)	
Depth of invasion				
T1	87	59 (67.8)	28 (32.2)	0.002
T2/3/4	115	52 (45.2)	63 (54.8)	
Lymph node metastasis				
Negative	113	74 (65.5)	39 (34.5)	0.001
Positive	89	37 (41.6)	52 (58.4)	
Stage				
I	106	72 (67.9)	34 (32.1)	<0.001
II/III	96	39 (40.4)	57 (59.4)	
Distant recurrence				
Negative	152	91 (59.9)	61 (40.1)	0.021
Positive	50	20 (40)	30 (60)	
Liver recurrence				
Negative	194	110 (56.7)	84 (43.3)	0.024
Positive	8	1 (12.5)	7 (87.5)	
Peritoneal recurrence				
Negative	182	99 (54.4)	83 (45.6)	0.814
Positive	20	12 (60)	8 (40)	
HER2				
Negative	186	107 (57.5)	79 (42.5)	0.017
Positive	16	4 (25)	12 (75)	

*P* < 0.05, statistically significant

### Correlation between EPHA3 expression and survival

**Fig. 2:**
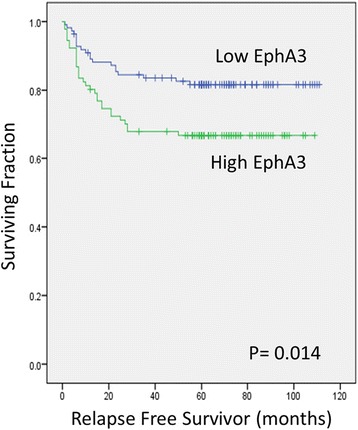
Relationship between EphA3 expression and gastric cancer patient relapse-free survival (RFS). Kaplan-Meier curves were plotted for RFS of low and high EphA3 expression in gastric cancer patients. *P* < 0.05, statistically significant

**Fig. 3:**
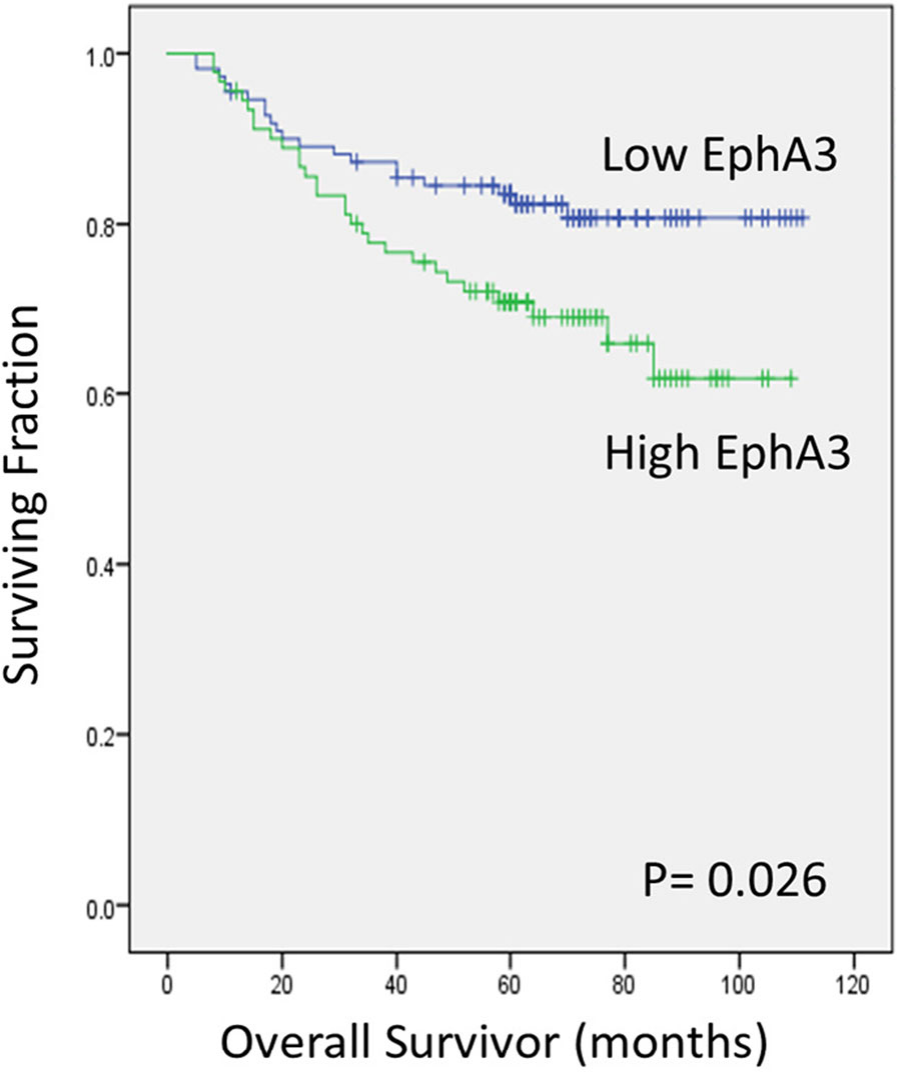
Relationship between EphA3 expression and gastric cancer patient overall survival (OS). Kaplan-Meier curves were plotted for OS of low and high EphA3 expression in gastric cancer patients. *P* < 0.05, statistically significant

Kaplan-Meier curves were plotted to assess the effect of EphA3 expression on relapse free survival (RFS) and overall survival (OS). Survival analysis by log-rank test suggested that high EphA3 expression associated with a significantly shorter RFS (82% vs 67%) and OS (82% vs 68.1%) (Fig. 2; Table 2). Multivariate analysis for RFS showed that depth of tumor [hazard ratio (HR) 9.3, 95% confidence interval (CI) 2.183–39.911, *p* = 0.003] and lymph node metastasis [hazard ratio (HR) 5.7, 95% confidence interval (CI) 2.349–13.997, *p* < 0.001] were independent prognostic factors (Table 2). Similarly multivariate analysis for overall survival (OS) also showed that depth of tumor [hazard ratio (HR) 8.8, 95% confidence interval (CI) 2.038–37.881, *p* = 0.004] and lymph node metastasis [hazard ratio (HR) 5.9, 95% confidence interval (CI) 2.417–14.537, *p* < 0.001] were independent prognostic factors for OS (Fig. 3, Table 3).

**Table 2:** Univariate (log-rank) and multivariate (Cox proportional-harzards) analyses of the association between relapse free survival and clinicopathological factors including EphA3 expression

Variables	Univariate (log-rank)		Multivariate		
	5-years RFS (%)	*p*	HR	95% CI	*p*
Age					
< 65	75.5	0.809			
≥ 65	74.5				
Gender					
Female	77.1	0.655			
Male	74.4				
Main location					
Middle or Lower	80.1	0.001	1		
Upper	55.8		1.684	0.937–3.029	0.082
WHO pathological type					
Differentiated	84	0.03	1		
Undifferentiated	66.3		1.616	0.841–3.103	0.149
Depth of invasion					
T1	97.7	<0.001	1		
T2,3,4	58.1		9.333	2.183–39.911	0.003
Lymph node metastasis					
Negative	94.7	<0.001	1		
Positive	50.5		5.734	2.349–13.997	<0.001
EPHA3					
Low	82.0	0.014	1		
High	67.0		1.313	0.705–2.447	0.391
HER2					
Negative	75.5	0.538			
Positive	68.8				

*RFS* relapse-free survival, *HR* harzard ratio, *CI* confidence interval; *P* < 0.05, statistically significant

**Table 3:** Univariate (log-rank) and multivariate (Cox proportional-harzards) analyses of the association between overalls survival and clinicopathological factors including EphA3 expression

Variables	Univariate (log-rank)			Multivariate	
	5-years OS (%)	*p*	HR	95% CI	*p*
Age					
< 65	76.5	0.578			
≥ 65	74.5				
Gender					
Female	79.2	0.516			
Male	74.4				
Main location					
Middle or Lower	80.1	0.001	1		
Upper	58.1		1.654	0.907–3.017	0.101
WHO pathological type					
Differentiated	83	0.01	1		
Undifferentiated	68.3		1.475	0.766–2.842	0.246
Depth of invasion					
T1	97.7	<0.001	1		
T2,3,4	59		8.785	2.038–37.881	0.004
Lymph node metastasis					
Negative	94.7	<0.001	1		
Positive	51.6		5.928	2.417–14.537	<0.001
EPHA3					
Low	82.0	0.026	1		
High	68.1		1.147	0.603–2.181	0.677
HER2					
Negative	75.76.15	0.431			
Positive	68.8				

*OS* overall survival, *HR* harzard ratio, *CI* confidence interval; *P* < 0.05; statistically significant

## Discussions

Our study suggests that high expression of EphA3 may play crucial roles in tumor development, metastasis, and survival in gastric cancer. To our best of knowledge, our study along with Xi et al is the only two articles regarding the clinical outcomes of the novel receptor EphA3 in gastric cancer [11].

EphA3 found abundantly in mesenchymal tissues of developing axial muscles, respiratory tract, kidney, and heart, is involved in mesoderm, neural patterning, and is crucial for endothelial-to-mesenchymal transition during heart development [12, 13]. There is very limited evidence for physiologic function, but EphA3 is overexpressed in solid and hematopoietic tumor cells [8, 11, 14]. The effect of EphA3 on human cancers is variable. High expression of EphA3 was related to lymph node metastasis and advanced stages in colorectal cancer [15] and was associated with higher Gleason score in prostate cancer [16]. In hepatocellular carcinoma, high EphA3 expression was related with tumor size, tumor grade, metastasis, venous invasion [9]. However, high expression of EphA3 was reported to suppress the growth of non-small cell lung cancer [10]. Eph receptor tyrosine kinases are crucial for intercellular communication during physiologic and oncogenic tissue patterning and tumor development [5, 17]. Differences in EphA3 are thought to generate certain morphological and biological characteristics, such as cell growth and viability, loss of cell adhesion to fibronectin, cell migration, and apoptosis. Abundant evidences show that aberrant regulation of EphA3 and its genetic variation are strongly related to the development and progression of many types of solid cancers [18–20]. Although the exact mechanism of how EphA3 regulates its downstream is not well understood, it is hypothesized that EphA subgroup stimulates tumor progression by activating Jak/Stat and Akt/PI3 K signals [21–24].

One of the limitations is that we do not have cell culture experiment results. Our study along with Xi et al [11] is the only two articles focusing on the newly discovered receptor EphA3. The recent study of Xi et al showed the higher expression of EphA3 in gastric adenocarcinoma than in normal tissue [11]. Although EphA3 failed to reach the statistical value for independent prognostic factor, our study showed that EphA3 overexpression was associated with depth of tumor, lymph node metastasis, stage, distant metastasis and recurrence of gastric cancer. These findings are in accord with Xi et al study. Other limitation is that our study mainly focused on immunohistochemical staining of EphA3. Further genetic evaluation or quantitative assessment is crucial to confirm the outcomes of this study, although mRNA expression level and Western blot of EphA3 was shown to be overexpressed in gastric cancer tissue than in normal tissue by Xi et al [11].

While Xi et al emphasized overexpression of EphA3 correlated with worse survival curve, our findings proved that high expression of EphA3 associated with poorer RFS of gastric cancer with higher rate of distant recurrence especially liver recurrence. Our study has suggested prospective investigation for the possibility of correlation between EphA3 and liver recurrence in gastric cancer.

## Conclusions

This present study showed that EphA3 overexpression was associated with depth of tumor, lymph node metastasis, stage, distant recurrence, liver recurrence and poorer RFS of gastric cancer. These findings hypothesize that EphA3 can be a potential target of molecular targeted therapy of gastric cancer.

## Abbreviations

CI: Confidence interval
DDW: Double-distilled water
EpHA3: Erythropoietin-producing hepatocellular A3
HER-2: Human epidermal growth factor receptor 2
HR: Hazard ratio
OS: Overall survival
PBS: Phosphate buffered saline
RFS: Relapse-free survival
RTKs: Receptor tyrosine kinases
TS-1: Titanium silicate
